# 骨髓红系比例≥50%的骨髓增生异常综合征患者临床特征及预后分析

**DOI:** 10.3760/cma.j.cn121090-20240517-00183

**Published:** 2024-07

**Authors:** 燕平 曾, 冰 李, 铁军 秦, 泽锋 徐, 士强 曲, 丽娟 潘, 清妍 高, 蒙 焦, 君颖 吴, 慧君 王, 承文 李, 玉娇 贾, 琦 孙, 志坚 肖

**Affiliations:** 1 中国医学科学院血液病医院（中国医学科学院血液学研究所），实验血液学国家重点实验室，国家血液系统疾病临床医学研究中心，细胞生态海河实验室，天津 300020 State Key Laboratory of Experimental Hematology, National Clinical Research Center for Blood Diseases, Haihe Laboratory of Cell Ecosystem, Institute of Hematology & Blood Diseases Hospital, Chinese Academy of Medical Sciences & Peking Union Medical College, Tianjin 300020, China; 2 天津医学健康研究院，天津 301600 Tianjin Institutes of Health Science, Tianjin 301600, China

**Keywords:** 骨髓增生异常综合征, 红系细胞, 环状铁粒幼红细胞, TP53基因, 预后, Myelodysplastic syndrome, Erythroid cells, Ring sideroblasts, TP53 gene, Prognosis

## Abstract

**目的:**

分析骨髓红系比例≥50％的骨髓增生异常综合征（MDS-E）患者临床和实验室特征及预后。

**方法:**

收集2014年5月至2023年6月于中国医学科学院血液病医院确诊的1 436例MDS初治患者病例资料，回顾性分析MDS-E患者临床特征、分子学等实验室特征以及总生存（OS）情况。

**结果:**

①MDS-E患者共337例（23.5％），与骨髓红系比例<50％的MDS（MDS-NE）患者相比，发病年龄、ANC、PLT更低（*P*值均<0.05）。MDS-E中诊断伴环状铁粒幼红细胞增多的MDS（MDS-RS）患者比例更高，多打击TP53基因突变检出率更高（*P*值均<0.05）。②在MDS-RS患者中，与MDS-NE相比，MDS-E组复杂染色体核型比例显著减低（0对11.9％，*P*＝0.048），TP53基因突变检出率更低（2.4％对15.1％，*P*＝0.053）。③在TP53突变的MDS患者中，MDS-E患者复杂染色体核型比例（87.5％对63.3％，*P*＝0.002）及多打击TP53突变检出率（84.0％对53.4％，*P*<0.001）均显著高于MDS-NE患者。④在MDS-RS患者中，MDS-E组的中位OS时间较MDS-NE组更长［未达到对63（95％*CI* 53～73）个月，*P*＝0.029］。在TP53突变且原始细胞增多的MDS患者中，MDS-E组中位OS时间较MDS-NE组更短［6（95％*CI* 2～10）个月对12（95％*CI* 9～15）个月，*P*＝0.022］。⑤多因素分析示，年龄≥65岁（*HR*＝2.47，95％*CI* 1.43～4.26，*P*＝0.001）、MCV≤100 fl（*HR*＝2.62，95％*CI* 1.54～4.47，*P*<0.001）、TP53突变（*HR*＝2.31，95％*CI* 1.29～4.12，*P*＝0.005）是MDS-E患者独立于修订的国际预后积分系统（IPSS-R）预后分层的不良预后因素。

**结论:**

MDS-RS患者中，MDS-E与更低的复杂染色体核型占比及TP53突变检出率相关，MDS-E组OS时间显著延长。TP53突变的MDS患者中，MDS-E与更高的复杂染色体核型占比和多打击TP53突变检出率相关，原始细胞增多伴TP53突变的患者中，MDS-E组OS时间显著缩短。年龄≥65岁、MCV≤100 fl及TP53突变是影响MDS-E患者生存的独立不良预后因素。

## Abstract (en)

**Objective:**

To analyze the clinical characteristics and prognosis of patients with myelodysplastic syndrome（MDS）with a bone marrow nucleated erythroid cell proportion of greater than or equal to 50％（MDS-E）.

**Methods:**

The clinical characteristics and prognostic factors of patients with MDS-E were retrospectively analyzed by collecting the case data of 1 436 newly treated patients with MDS diagnosed in the Institute of Hematology and Blood Diseases Hospital, Chinese Academy of Medical Sciences from May 2014 to June 2023.

**Results:**

A total of 1 436 newly diagnosed patients with complete data were included in the study, of which 337（23.5％）patients with MDS-E had a younger age of onset and lower neutrophil and platelet counts compared with those in patients with an erythroid cell proportion of less than 50％（MDS-NE）（all *P*<0.05）. The proportion of MDS cases with ring sideroblasts（MDS-RS）was higher in the MDS-E group than in the MDS-NE group, and multi-hit TP53 mutations were more enriched in the MDS-E group than in the MDS-NE group（all *P*<0.05）. Among patients with MDS-RS, the frequency of complex karyotypes and the TP53 mutation rate were significantly lower in the MDS-E group than in the MDS-NE group（0 *vs* 11.9％, *P*＝0.048 and 2.4％ *vs* 15.1％, *P*＝0.053, respectively）. Among patients with TP53 mutations, the frequencies of complex karyotypes and multi-hit TP53 mutations were significantly higher in the MDS-E group than in the MDS-NE group（87.5％ *vs* 64.6％, *P*＝0.003 and 84.0％ *vs* 54.2％, *P*<0.001, respectively）. Survival analysis of patients with MDS-RS found that the overall survival（OS）in the MDS-E group was better than that in the MDS-NE group［not reached *vs* 63（95％ *CI* 53.3–72.7）months, *P*＝0.029］. Among patients with TP53 mutations and excess blasts, the OS in the MDS-E group was worse than that in the MDS-NE group［6（95％ *CI* 2.2–9.8）months *vs* 12（95％ *CI* 8.9–15.1）months, *P*＝0.022］. Multivariate analysis showed that age of ≥65 years（*HR*＝2.47, 95％ *CI* 1.43–4.26, *P*＝0.001）, mean corpuscular volume（MCV）of ≤100 fl（*HR*＝2.62, 95％ *CI* 1.54–4.47, *P*<0.001）, and TP53 mutation（*HR*＝2.31, 95％ *CI* 1.29–4.12, *P*＝0.005）were poor prognostic factors independent of the Revised International Prognostic Scoring System（IPSS-R）prognosis stratification in patients with MDS-E.

**Conclusion:**

Among patients with MDS-RS, MDS-E was strongly associated with a lower proportion of complex karyotypes and TP53 mutations, and the OS in the MDS-E group was longer than that in the MDS-NE group. Among patients with TP53 mutations, MDS-E was strongly associated with complex karyotypes and multi-hit TP53 mutations, and among TP53-mutated patients with excess blasts, the OS in the MDS-E group was shorter than that in the MDS-NE group. Age of ≥65 years, MCV of ≤100 fl, and TP53 mutation were independent adverse prognostic factors affecting OS in patients with MDS-E.

骨髓增生异常综合征（myelodysplastic syndrome, MDS）是一组异质性起源于造血干/祖细胞的髓系肿瘤，表现为外周血血细胞计数减少、骨髓和外周血一系或多系发育异常及高风险向急性髓系白血病（acute myeloid leukemia, AML）转化[1]。红系发育异常是MDS各亚型中最为常见的血细胞发育异常。除了核出芽、核间桥、核碎裂、多核、核过分叶，核的巨幼红细胞样改变、环状铁粒幼红细胞、胞质空泡、PAS阳性等均属红系发育异常的细胞形态学异常改变，红系细胞占骨髓有核细胞的比例异常>60％或<5％亦归于红系发育异常[2]。

1976年法、美、英（French-American-British, FAB）协作组定义的AML-M6型[3]（主要标准是骨髓红系≥50％，原始粒细胞+早幼粒细胞>30％），按WHO（2016）标准[4]诊断，大多数患者改诊为MDS，使得红系比例异常增高的MDS患者群体进一步扩大。迄今尚无骨髓红系比例≥50％ MDS患者（MDS-E）的相关研究报道。本研究我们回顾性分析了我院MDS-E患者的临床特征、分子学等实验室特征以及总生存情况，现报道如下。

## 病例与方法

1. 病例资料：2014年5月至2023年6月于中国医学科学院血液病医院MDS和骨髓增殖性肿瘤（MPN）诊疗中心确诊、具有完整病历资料的1 436例MDS初治患者纳入研究，所有患者均按WHO（2016）标准[4]诊断，并按WHO（2022）标准[5]和国际共识分类（International Consensus Classification, ICC）标准[6]进行重新诊断分型。

2. 染色体核型分析：短期培养法常规制备染色体标本，采用R显带法分析核型，根据《人类细胞遗传学国际命名体制（ISCN2016）》描述核型，按照修订的国际预后积分系统（IPSS-R）对染色体核型进行预后分组[7]。

3. 二代测序检测基因突变：分离患者骨髓单个核细胞，使用PCR引物扩增目的基因组，将目标区域DNA富集后，采用Ion Torrent测序平台进行测序。测序后原始数据利用CCDS、HG19、dbSNP（v138）、1000genomes、COSMIC、PolyPhen-2等数据库进行生物信息学分析，筛选致病性基因突变位点。具体方法参见本研究组此前已发表文献[8]–[9]。

4. 随访及相关定义：随访截止时间为2023年9月16日，随访资料来源于住院病历、门诊病历及电话随访记录。对随访期间死亡的病例，按照病历记录或与患者家属电话联系确认。中位随访时间为25（9～52）个月，共123例（8.6％）患者失访。总体生存（overall survival, OS）时间指自诊断日期到死亡或造血干细胞移植或末次随访日期。根据WHO（2022）标准，骨髓原始细胞<5％或外周血原始细胞<2％定义为低原始细胞（LB），骨髓原始细胞≥5％或外周血原始细胞≥2％或出现Auer小体定义为原始细胞增多（IB）。

5. 统计学处理：统计分析利用SPSS 26.0完成。非正态分布的计量资料以“中位数（四分位数间距）”表示，采用Mann-Whitney *U*检验进行组间比较；率的比较采用卡方检验或Fisher精确概率法。采用Kaplan-Meier法绘制生存曲线，Cox比例风险回归模型进行预后因素分析，预后因素分析中将连续变量转化为二分类变量，年龄参照欧美发达国家老年人的标准以65岁为界，平均红细胞体积（MCV）以100 fl为界。所有检验均为双侧。应用Graphpad Prism 9.0及R 4.3.2绘图。

## 结果

一、MDS-E患者临床特征

1. 患者一般特征：1 436例MDS患者中，MDS-E患者共337例（23.5％），其发病年龄显著低于MDS-NE患者。MDS-E与MDS-NE患者相比，ANC、PLT、骨髓纤维化2～3级比例、血清促红细胞生成素（erythropoietin，EPO）水平较低，骨髓巨核系发育异常比例较高，差异均有统计学意义（均*P*<0.05）（表1）。性别、HGB水平、MCV、粒系发育异常比例等两组差异均无统计学意义。此外，两组患者无论是按照WHO（2016）（*P*＝0.001），还是WHO（2022）（*P*<0.001）或ICC（*P*＝0.003）标准诊断分型的亚型构成占比差异均有统计学意义。根据WHO（2016）标准诊断，MDS-E患者中伴环状铁粒幼红细胞增多的MDS（MDS-RS）比例显著高于MDS-NE患者［42例（12.5％）对73例（6.6％），*P*＝0.001］；根据WHO（2022）标准诊断，MDS-E患者中伴SF3B1突变的MDS（MDS-SF3B1）［39例（11.6％）对73例（6.6％），*P*＝0.005］及伴双等位TP53基因突变的MDS（MDS-biTP53）［42例（12.5％）对98例（8.9％），*P*＝0.055］的比例高于MDS-NE；同样，根据ICC标准诊断，MDS-E患者中MDS-SF3B1［31例（9.2％）对56例（5.1％），*P*＝0.006］及伴TP53突变的MDS（MDS-TP53）［34例（10.1％）对50例（4.5％），*P*<0.001］比例均显著高于MDS-NE。

**表1:** 不同骨髓红系比例分组的骨髓增生异常综合征（MDS）患者一般临床特征

临床特征	全部（1436例）	MDS-E（337例）	MDS-NE（1099例）	统计量	*P*值
男性[例（%）]	920（64.1）	229（68.0）	691（62.9）	*χ^2^*=2.89	0.089
年龄[岁，*M*（*IQR*）]	55（43~63）	52（41~61）	56（44~64）	*z*=3.28	0.001
HGB[g/L，*M*（*IQR*）]	79（66~96）	78（67~93）	80（66~98）	*z*=1.61	0.108
ANC[×10^9^/L，*M*（*IQR*）]	1.09（0.64~1.95）	0.99（0.60~1.72）	1.15（0.66~2.06）	*z*=3.18	0.001
PLT[×10^9^/L，*M*（*IQR*）]	62（32~126）	58（28~104）	63（33~132）	*z*=2.27	0.023
网织红细胞百分比[%，*M*（*IQR*）]	1.88（1.12~2.95）	2.56（1.70~4.17）	1.67（1.01~2.57）	*z*=10.52	<0.001
MCV[fl，*M*（*IQR*）]	100（93~107）	100.5（93.5~107.5）	99.6（92.9~107.4）	*z*=0.86	0.391
骨髓原始细胞比例[%，*M*（*IQR*）]	3（1~7）	2（1~6）	3（1~8）	*z*=3.47	0.001
LDH[U/L，*M*（*IQR*）]	213（168~276）	228（173~321）	209（166~267）	*z*=3.82	<0.001
血清铁蛋白[µg/L，*M*（*IQR*）]	430（219~806）	392（202~744）	447（226~833）	*z*=2.00	0.045
血清EPO[U/L，*M*（*IQR*）]	255（70~758）	176（61~729）	302（73~758）	*z*=2.53	0.011
环状铁粒幼红细胞[%，*M*（*IQR*）]	0（0~2）	0（0~7）	0（0~1）	*z*=4.38	<0.001
红系发育异常[例（%）]	933（65.0）	303（89.9）	630（57.3）	*χ^2^*=120.34	<0.001
粒系发育异常[例（%）]	918（63.9）	206（61.1）	712（64.8）	*χ^2^*=1.50	0.221
巨核系发育异常[例（%）]	1140（80.2）	280（83.8）	860（79.0）	*χ^2^*=3.69	0.055
骨髓纤维化2~3级[例（%）]	181（12.8）	22（6.7）	159（14.7）	*χ^2^*=14.05	<0.001

**注** MDS-E：骨髓红系比例≥50%的MDS；MDS-NE：骨髓红系比例<50％的MDS；MCV：平均红细胞体积；LDH：乳酸脱氢酶；EPO：促红细胞生成素

2. 患者细胞遗传学特征：MDS-E患者中有311例患者有可分析的染色体核型，其中异常染色体核型检出率为53.1％（191/311），复杂核型检出率为18.3％（57/311），异常核型中+8最常见（52例，16.7％），其次是20q−（47例，15.1％）、−5/5q−（35例，11.3％）、−7/7q−（25例，8.0％）。根据IPSS-R细胞遗传学预后分组，染色体预后较差（差/极差组）的MDS-E患者共62例（19.9％）。根据IPSS-R预后分组，预后较高危组（高危/极高危组）的MDS-E患者共127例（40.8％）。MDS-E与MDS-NE患者相比，MDS-E患者中20q−异常检出率更高［47例（15.1％）对85例（8.7％），*P*＝0.001］，而−5/5q−、−7/7q−、−17/17p−、+8、复杂核型异常检出率，IPSS-R细胞遗传学分组，IPSS-R预后分组两组间差异均无统计学意义。

3. 患者分子遗传学特征：337例MDS-E患者进行了二代测序检测，基因突变图谱见图1。其中检出率较高的依次为U2AF1（82例，24.3％）、TP53（50例，14.8％）、ASXL1（49例，14.5％）、SF3B1（40例，11.9％）、RUNX1（33例，9.8％）、DNMT3A（31例，9.2％）、TET2（27例，8.0％）和BCOR（25例，7.4％）。与MDS-NE患者相比，MDS-E患者多打击TP53［42例（12.5％）对78例（7.1％），*P*＝0.002］、BCOR［25例（7.4％）对41例（3.7％），*P*＝0.005］、STAG2［15例（4.5％）对26例（2.4％），*P*＝0.044］基因突变检出率显著增高，ASXL1［49例（14.5％）对225例（20.5％），*P*＝0.015］、SRSF2［8例（2.4％）对64例（5.8％），*P*＝0.011］、ZRSR2［1例（0.3％）对34例（3.1％），*P*＝0.004］基因突变检出率显著减低（图2）。

**图1:**
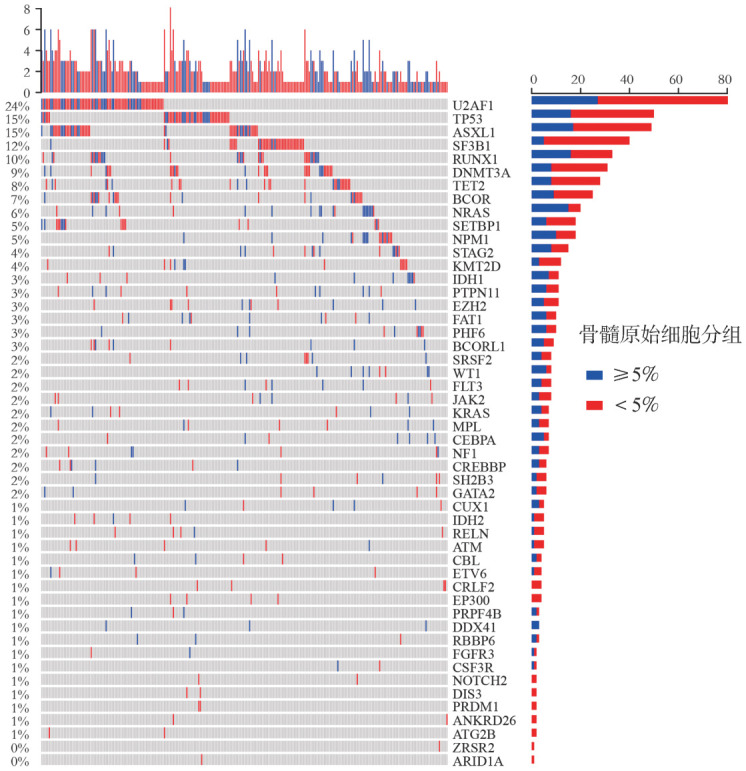
MDS-E患者基因突变谱系特征 **注** MDS-E：骨髓红系比例≥50％的骨髓增生异常综合征

**图2:**
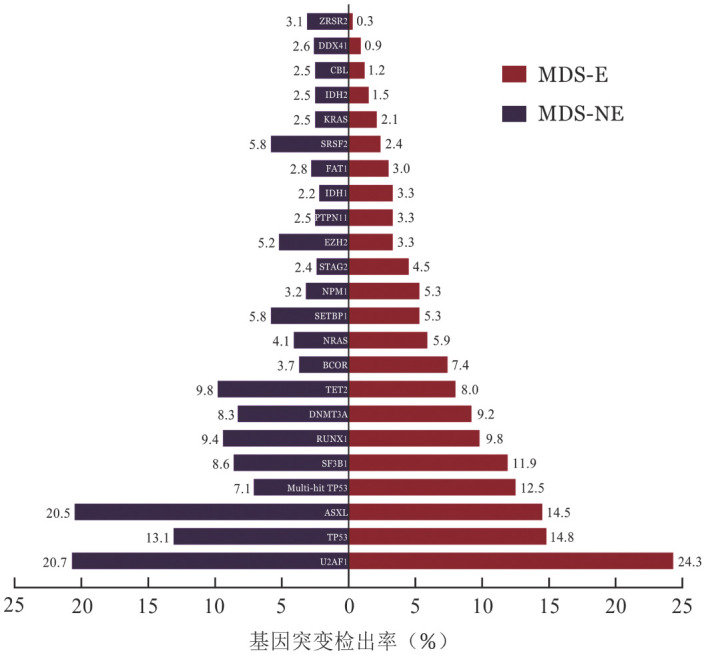
MDS-E和MDS-NE患者基因突变检出率比较 **注** MDS-E：骨髓红系比例≥50％的骨髓增生异常综合征；MDS-NE：骨髓红系比例<50％的骨髓增生异常综合征；Multi-hit TP53：多打击TP53突变

根据骨髓原始细胞比例将MDS-E患者分成2组，骨髓原始细胞≥5％组患者RUNX1［16例（16.0％）对17例（7.2％），*P*＝0.013］、NRAS［15例（15.0％）对5例（2.1％），*P*<0.001］、NPM1［10例（10.0％）对8例（3.4％），*P*＝0.013］、IDH1［7例（7.0％）对4例（1.7％），*P*＝0.030］基因突变检出率明显高于骨髓原始细胞<5％组，SF3B1基因突变检出率明显低于骨髓原始细胞<5％组［5例（5.0％）对35例（14.8％），*P*＝0.011］。

以上结果表明MDS-E患者中MDS-RS比例及多打击TP53突变检出率更高。因此，我们对MDS-RS及伴TP53突变的MDS患者进行亚组分析。

二、MDS-RS患者中MDS-E与MDS-NE的临床和实验室特征

MDS-RS患者共115例，其中MDS-E患者42例（36.5％），MDS-NE患者73例（63.5％），MDS-E组患者复杂核型的检出率显著低于MDS-NE患者（0对11.9％，*P*＝0.048），IPSS-R染色体预后差和极差组比例也低（0对13.4％，*P*＝0.045），IPSS-R预后较高危组比例低（2.6％对14.9％，*P*＝0.054）。而性别、年龄、外周血细胞计数、网织红细胞百分比、MCV、LDH、血清EPO、血清铁蛋白、环状铁粒幼红细胞、三系发育异常比例、骨髓纤维化2～3级比例等两组间差异均无统计学意义。在分子遗传学特征方面，MDS-RS患者中，MDS-E组患者TP53基因突变检出率低于MDS-NE组（2.4％对15.1％，*P*＝0.053），而SF3B1基因突变检出率差异无统计学意义（表2）。

**表2:** MDS-RS患者中MDS-E与MDS-NE临床和实验室特征比较

临床特征	全部（115例）	MDS-E（42例）	MDS-NE（73例）	统计量	*P*值
男性[例（%）]	66（57.4）	28（66.7）	38（52.1）	χ^2^=2.33	0.127
年龄[岁，*M*（*IQR*）]	59（51~66）	56（49~66）	59（52~68）	*z*=1.22	0.222
HGB[g/L，*M*（*IQR*）]	74（65~91）	73（63~89）	76（69~93）	*z*=1.43	0.153
ANC[×10^9^/L，*M*（*IQR*）]	1.84（1.16~2.74）	1.86（1.14~2.67）	1.84（1.26~2.97）	*z*=0.99	0.319
PLT[×10^9^/L，*M*（*IQR*）]	158（73~249）	164（98~261）	155（68~243）	*z*=0.63	0.530
网织红细胞百分比[%，*M*（*IQR*）]	1.65（1.04~2.80）	1.91（1.11~3.39）	1.61（0.94~2.61）	*z*=1.45	0.146
MCV[fl，*M*（*IQR*）]	103.6（96.3~110.3）	104.1（96.3~113.0）	103.5（96.2~109.6）	*z*=0.95	0.344
骨髓原始细胞比例[%，*M*（*IQR*）]	1（0~2）	0.5（0~1）	1（0~2）	*z*=1.92	0.055
LDH[U/L，*M*（*IQR*）]	199（161~261）	181（154~221）	208（173~274）	*z*=1.77	0.077
血清铁蛋白[µg/L，*M*（*IQR*）]	629（372~1142）	717（417~1241）	541（342~1070）	*z*=0.76	0.449
血清EPO[U/L，*M*（*IQR*）]	384（101~758）	447（91~762）	320（104~755）	*z*=0.54	0.591
环状铁粒幼红细胞[%，*M*（*IQR*）]	28（18~48）	30（20~52）	26（16~44）	*z*=1.33	0.184
红系发育异常[例（%）]	98（85.2）	41（97.6）	57（78.1）	χ^2^=8.08	0.004
粒系发育异常[例（%）]	65（56.5）	23（54.8）	42（57.5）	χ^2^=0.08	0.773
巨核系发育异常[例（%）]	81/113（71.7）	32/42（76.2）	49/71（69.0）	χ^2^=0.67	0.413
骨髓纤维化2~3级[例（%）]	12/114（10.5）	2/42（4.8）	10/72（13.9）	χ^2^=1.48	0.224
染色体核型[阳性例数/总例数（%）]					
−5/5q−	1/105（1.0）	0/38（0.0）	1/67（1.5）	Fisher	1.000
−7/7q−	3/105（2.9）	1/38（2.6）	2/67（3.0）	Fisher	1.000
+8	16/105（15.2）	6/38（15.8）	10/67（14.9）	χ^2^=0.01	0.906
20q−	13/105（12.4）	6/38（15.8）	7/67（10.4）	χ^2^=0.24	0.624
复杂核型	8/105（7.6）	0/38（0.0）	8/67（11.9）	Fisher	0.048
正常核型	58/105（55.2）	23/38（60.5）	35/67（52.2）	χ^2^=0.67	0.412
IPSS-R染色体分组[例（%）]				χ^2^=4.00	0.045
极好/好/中等	96（91.4）	38（100.0）	58（86.6）		
差/极差	9（8.6）	0（0.0）	9（13.4）		
IPSS-R预后分组[例（%）]				Fisher	0.054
极低/低/中危	94（89.5）	37（97.4）	57（85.1）		
高危/极高危	11（10.5）	1（2.6）	10（14.9）		
基因突变[例（%）]					
SF3B1	71（61.7）	28（66.7）	43（58.9）	χ^2^=0.68	0.410
ASXL1	22（19.1）	11（26.2）	11（15.1）	χ^2^=2.13	0.144
TET2	17（14.8）	4（9.5）	13（17.8）	χ^2^=1.45	0.228
U2AF1	14（12.2）	4（9.5）	10（13.7）	χ^2^=0.44	0.510
TP53	12（10.4）	1（2.4）	11（15.1）	Fisher	0.053
RUNX1	9（7.8）	5（11.9）	4（5.5）	χ^2^=0.77	0.382
DNMT3A	9（7.8）	5（11.9）	4（5.5）	χ^2^=0.77	0.382
SETBP1	7（6.1）	3（7.1）	4（5.5）	Fisher	0.705
EZH2	5（4.3）	1（2.4）	4（5.5）	Fisher	0.651
Muliti-TP53	4（3.5）	0（0.0）	4（5.5）	Fisher	0.295
SRSF2	4（3.5）	1（2.4）	3（4.1）	Fisher	1.000
CBL	3（2.6）	2（4.8）	1（1.4）	Fisher	0.553

**注** MDS：骨髓增生异常综合征；MDS-RS伴环状铁粒幼红细胞增多的MDS；MDS-E：骨髓红系比例≥50%的MDS；MDS-NE：骨髓红系比例<50%的MDS；MCV：平均红细胞体积；LDH：乳酸脱氢酶；EPO：促红细胞生成素；IPSS-R：修订的国际预后积分系统；Multi-hit TP53：多打击TP53突变

三、伴TP53突变MDS患者中MDS-E与MDS-NE的临床和实验室特征

共195例患者检出TP53基因突变，其中MDS-E患者50例（25.6％），MDS-NE患者145例（74.4％），两组比较发现MDS-E组患者年龄更小、血清EPO水平更低，而网织红细胞百分比、LDH水平均显著增高，复杂染色体核型的比例也显著高于MDS-NE患者（87.5％对63.3％，*P*＝0.002）。性别、外周血三系血细胞计数、MCV、血清铁蛋白、环状铁粒幼红细胞、粒系/巨核系发育异常、骨髓纤维化2～3级比例、IPSS-R预后分组差异均无统计学意义。在分子遗传学特征方面，MDS-E组患者中多打击TP53突变检出率显著增高（84.0％对53.4％，*P*<0.001），其他共突变检出率差异无统计学意义（均*P*>0.05）（表3）。

**表3:** 伴TP53突变的MDS患者中MDS-E与MDS-NE临床和实验室特征比较

临床特征	全部（195例）	MDS-E（50例）	MDS-NE（145例）	统计值	*P*值
男性[例（%）]	133（68.6）	38（76.0）	95（66.0）	*χ*^2^=1.73	0.188
年龄[岁，*M*（*IQR*）]	61（53~66）	58（46~63）	62（55~68）	*z*=3.52	<0.001
HGB[g/L，*M*（*IQR*）]	75（64~88）	77（64~91）	75（65~88）	*z*=0.09	0.931
ANC[×10^9^/L，*M*（*IQR*）]	1.09（0.55~1.84）	1.02（0.64~2.20）	1.13（0.52~1.77）	*z*=0.31	0.755
PLT[×10^9^/L，*M*（*IQR*）]	51（26~89）	44（25~75）	53（27~89）	*z*=0.75	0.452
网织红细胞百分比[%，*M*（*IQR*）]	1.49（0.73~2.23）	1.90（1.07~4.15）	1.40（0.63~1.98）	*z*=3.11	0.002
MCV[fl，*M*（*IQR*）]	97.1（90.4~104.4）	96.0（89.9~101.7）	97.8（90.4~104.8）	*z*=1.56	0.119
骨髓原始细胞比例[%，*M*（*IQR*）]	4.5（1.5~10.0）	3（1~6）	6（2~11）	*z*=3.20	0.001
LDH[U/L，*M*（*IQR*）]	223（166~359）	296（187~438）	212（164~307）	*z*=2.56	0.011
血清铁蛋白[µg/L，*M*（*IQR*）]	444（288~794）	440（287~733）	447（288~801）	*z*=0.11	0.912
血清EPO[U/L，*M*（*IQR*）]	130（42~444）	117（30~178）	153（52~521）	*z*=3.15	0.032
环状铁粒幼红细胞[%，*M*（*IQR*）]	2（0~13）	5（1~14）	2（0~12）	*z*=1.50	0.134
红系发育异常[例（%）]	137（70.6）	48（96.0）	89（61.8）	*χ*^2^=20.92	<0.001
粒系发育异常[例（%）]	103（53.1）	22（44.0）	81（56.3）	*χ*^2^=2.24	0.135
巨核系发育异常[例（%）]	170（88.5）	46（93.9）	124（86.7）	*χ*^2^=1.85	0.174
骨髓纤维化2~3级[例（%）]	47（24.7）	11（22.9）	36（25.4）	*χ*^2^=0.11	0.735
染色体核型[阳性例数/总例数（%）]					
−5/5q−	99/175（56.6）	31/48（64.6）	68/127（53.5）	*χ*^2^=1.73	0.189
−7/7q−	62/175（35.4）	19/48（39.6）	43/127（33.9）	*χ*^2^=0.50	0.480
−17/17p−	46/175（26.3）	11/48（22.9）	35/127（27.6）	*χ*^2^=0.39	0.534
+8	27/175（15.4）	9/48（18.8）	18/127（14.2）	*χ*^2^=0.56	0.455
20q−	32/175（18.3）	13/48（27.1）	19/127（15.0）	*χ*^2^=3.43	0.064
复杂核型	124/175（70.9）	42/48（87.5）	82/127（64.6）	*χ*^2^=8.87	0.003
正常核型	24/175（13.7）	3/48（6.3）	21/127（16.5）	*χ*^2^=3.11	0.078
IPSS-R染色体分组[例（%）]				*χ*^2^=7.26	0.007
极好/好/中等	48（27.3）	6（12.5）	42（32.8）		
差/极差	128（72.7）	42（87.5）	86（67.2）		
IPSS-R预后分组[例（%）]				*χ*^2^=2.49	0.114
极低/低/中危	40（22.7）	7（14.6）	33（25.8）		
高危/极高危	136（77.3）	41（85.4）	95（74.2）		
基因突变[例（%）]					
Muliti-hit TP53	120（61.9）	42（84.0）	78（54.2）	*χ*^2^=14.00	<0.001
U2AF1	24（12.4）	6（12.0）	18（12.5）	*χ*^2^=0.01	0.926
DNMT3A	22（11.3）	7（14.0）	15（10.4）	*χ*^2^=0.47	0.491
ASXL1	19（9.8）	3（6.0）	16（11.1）	*χ*^2^=0.60	0.440
TET2	16（8.2）	4（8.0）	12（8.3）	Fisher	1.000
SF3B1	16（8.2）	3（6.0）	13（9.0）	*χ*^2^=0.14	0.710
EP300	12（6.2）	2（4.0）	10（6.9）	*χ*^2^=0.16	0.686
SETBP1	8（4.1）	2（2.0）	6（4.2）	Fisher	1.000
RUNX1	7（3.6）	2（4.0）	5（3.5）	Fisher	1.000
EZH2	7（3.6）	3（6.0）	4（2.8）	*χ*^2^=0.38	0.540
BCOR	7（3.6）	3（6.0）	4（2.8）	*χ*^2^=0.38	0.540
DDX41	6（3.1）	0（0.0）	6（4.2）	*χ*^2^=0.98	0.321
PTPN11	5（2.6）	1（2.0）	4（2.8）	Fisher	1.000
SRSF2	4（2.1）	0（0.0）	4（2.8）	*χ*^2^=0.38	0.540

**注** MDS：骨髓增生异常综合征；MDS-E：骨髓红系比例≥50%的MDS；MDS-NE：骨髓红系比例<50%的MDS；MCV：平均红细胞体积；LDH：乳酸脱氢酶；EPO：促红细胞生成素；IPSS-R：修订的国际预后积分系统；Multi-hit TP53：多打击TP53突变

四、生存分析

在全部患者中，MDS-E与MDS-NE两组患者中位OS时间分别为未达到和49（95％C*I* 33～57）个月（*P*＝0.064）。进一步行亚组分析：MDS-RS患者中，MDS-E中位OS时间显著长于MDS-NE患者［未达到对63（95％*CI* 53～73）个月，*P*＝0.029］（图3A）。而伴TP53突变的MDS中，MDS-E与MDS-NE患者中位OS时间差异无统计学意义［11（95％*CI* 7～15）个月对15（95％*CI* 11～19）个月，*P*＝0.202］（图3B）。LB且伴TP53突变患者中，MDS-E与MDS-NE相比中位OS时间差异无统计学意义［13（95％*CI* 8～18）个月对25（95％*CI* 11～39）个月，*P*＝0.421］（图3C），但在IB且伴TP53突变患者中，MDS-E患者中位OS时间为6（95％*CI* 2～10）个月，明显短于MDS-NE患者的12（95％*CI* 9～15）个月（*P*＝0.022）（图3D）。而在IB且不伴TP53突变的MDS患者中比较MDS-E和MDS-NE两组的中位OS时间差异无统计学意义［27（95％*CI* 6～48）个月对26（95％*CI* 21～31）个月，*P*＝0.545］。

**图3:**
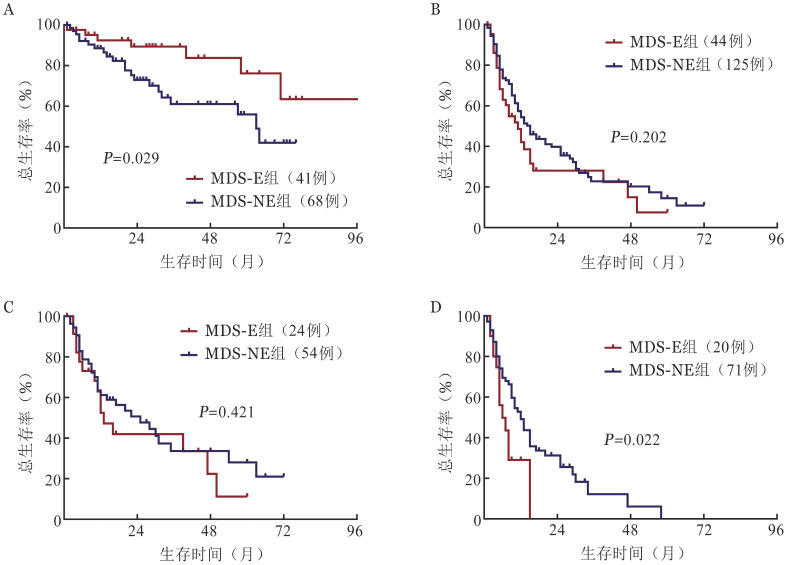
MDS-E与MDS-NE患者总生存曲线比较 **A** MDS-RS亚组；**B** 伴TP53突变的MDS亚组；**C** LB且伴TP53突变亚组；**D** IB且伴TP53突变 **注** MDS：骨髓增生异常综合征；MDS-E：骨髓红系比例≥50％的MDS；MDS-NE：骨髓红系比例<50％的MDS；MDS-RS：伴环状铁粒幼红细胞增多的MDS；LB：骨髓原始细胞<5％或外周血原始细胞<2％；IB：骨髓原始细胞≥5％或外周血原始细胞≥2％或出现Auer小体

五、MDS-E患者的预后影响因素分析

在全部MDS-E患者中，将年龄、性别、MCV、IPSS-R预后分层及可能影响生存的基因突变等因素分别行Cox单因素分析，随后将单因素分析中*P*<0.1的因素纳入Cox多因素分析，结果显示年龄≥65岁（*HR*＝2.47，95％*CI* 1.43～4.26，*P*＝0.001）、MCV≤100 fl（*HR*＝2.62，95％*CI* 1.54～4.47，*P*<0.001）、TP53突变（*HR*＝2.31，95％*CI* 1.29～4.12，*P*＝0.005）均是MDS-E患者独立于IPSS-R预后分组的不良预后因素（表4）。

**表4:** 影响MDS-E患者总生存的单因素和多因素分析

变量	单因素分析		多因素分析	
	*HR*（95%*CI*）	*P*值	*HR*（95%*CI*）	*P*值
年龄≥65岁	1.77（1.08~2.89）	0.023	2.47（1.43~4.26）	0.001
女性	0.85（0.54~1.35）	0.501		
IPSS-R非极低危	2.19（1.75~2.73）	<0.001	1.77（1.39~2.26）	<0.001
MCV≤100 fl	3.35（2.13~5.28）	<0.001	2.62（1.54~4.47）	<0.001
U2AF1突变	1.09（0.66~1.82）	0.733		
TP53突变	5.29（3.36~8.35）	<0.001	2.31（1.29~4.12）	0.005
SF3B1突变	1.58（0.84~2.98）	0.157		
ASXL1突变	0.81（0.42~1.56）	0.526		
RUNX1突变	1.31（0.68~2.53）	0.424		
DNMT3A突变	1.53（0.81~2.88）	0.189		
TET2突变	2.10（1.16~3.79）	0.014	1.46（0.76~2.78）	0.256
BCOR突变	1.23（0.53~2.83）	0.624		

**注** MDS-E：骨髓红系比例≥50％的骨髓增生异常综合征；MCV：平均红细胞体积；IPSS-R：修订的国际预后积分系统

## 讨论

本研究我们发现，相较于MDS-NE患者，MDS-E患者发病相对年轻，中位年龄52岁，并且MDS-E患者有更高的网织红细胞百分比和更低的ANC、PLT，与Wang等[10]结果基本一致，但本研究结果差异更显著，归因于本研究MDS-E中包含部分改诊前的红白血病患者。在诊断分型方面，MDS-E患者中MDS-RS比例占比明显高于MDS-NE，与Bennett等[11]结果基本一致，这主要与MDS-RS发病机制有关[12]。在分子遗传学方面，MDS-E患者中TP53突变检出率较高，其中多打击TP53突变检出率明显高于MDS-NE组，而既往的研究在未纳入红白血病患者时比较无显著差异[10]，这可能与红白血病患者中较高的TP53突变检出率有关[13]。但TP53突变与红系增生的关系目前尚不明确，TP53突变可能通过异常的细胞周期调控导致红系分化发育异常[14]–[15]。基于上述发现，我们将与红细胞比例显著增高密切相关的MDS-RS和伴TP53突变亚组进行了分析。

MDS-RS患者中，MDS-E组OS时间显著延长，可能与更低的原始细胞比例、复杂核型比例和TP53突变检出率有关，也有可能是由于这组患者对EPO治疗反应更佳，但目前尚无相关文献支持，有待进一步研究。目前关于TP53在MDS中的预后研究主要集中在不同等位基因状态、突变负荷等的比较[16]–[17]，其主要的观点是多打击TP53突变或TP53突变负荷越高的患者生存更差。尚未有研究报道红系增生水平在TP53突变患者中的意义。本研究显示，在伴TP53突变患者中，MDS-E组生存相对更差，可能与其更高的复杂核型占比及多打击TP53检出率有关，特别是在伴原始细胞增多患者中，生存差异更显著，可能和该患者群体中包含较多改诊前诊断红白血病的MDS患者相关。Mashima等[18]也曾报道，在骨髓原始细胞≥5％的MDS患者中MDS-E相较于MDS-NE OS期明显缩短，而在骨髓原始细胞<5％的MDS患者中OS期无明显差异。因此，在原始细胞增多的MDS患者中，尤其是伴TP53基因突变的患者，红系增生明显活跃是否提示不良预后值得进一步讨论。

对MDS-E患者预后行多因素分析发现，年龄≥65岁或IPSS-R预后积分更高均为MDS-E患者独立的不良预后因素，这一结果与既往文献报道一致[7],[19]。而TET2突变在单因素分析中为不良预后因素，再纳入年龄和其他因素分析后不再是预后危险因素，这可能与老年群体中存在相对较高的TET2突变发生率有关[20]–[21]。史仲珣等[22]发现在骨髓原始细胞<5％的MDS患者中，MCV≤100 fl组相较于MCV>100 fl组患者中位OS时间显著缩短，本研究中MCV≤100 fl无论在单因素分析还是纳入基因突变和其他临床指标的多因素分析中均是MDS-E患者不良预后因素。与既往多中心研究结果一致[16],[23]–[24]，TP53突变同样为MDS-E患者独立于IPSS-R的不良预后因素。

综上，本研究我们发现骨髓红系比例≥50％在不同的MDS患者中临床特征及预后意义不同。红系比例≥50％在MDS-RS患者中常提示更低危的表型及更佳的预后。相反，在TP53突变患者中，红系比例≥50％与更高危的临床特征密切相关，在原始细胞增多的TP53突变患者中，红系比例≥50％往往提示更差的预后。本研究存在以下不足：①作为单中心、回顾性研究，可能存在偏倚造成结果误差；②由于MDS-RS患者接受EPO的剂量、疗程、依从性等差异较大，因此无法对红系比例≥50％ MDS-RS患者应用这类药物治疗的疗效进行准确评价。本研究结论有待全国多中心、前瞻性临床试验验证。
